# 1-{(*Z*)-[(2,3-Dihy­droxy­prop­yl)amino]­methyl­idene}naphthalen-2(1*H*)-one

**DOI:** 10.1107/S1600536812051070

**Published:** 2012-12-22

**Authors:** Mehmet Akkurt, Peter N. Horton, Shaaban K. Mohamed, Antar A. Abdelhamid, Mahmoud A. A. El Remaily

**Affiliations:** a Department of Physics, Faculty of Sciences, Erciyes University, 38039 Kayseri, Turkey; b School of Chemistry, University of Southampton, Highfield, Southampton SO17 1BJ, England; c Chemistry and Environmental Division, Manchester Metropolitan University, Manchester M1 5GD, England; d Chemistry Department, Faculty of Science, Minia University, El-Minia, Egypt; e Department of Chemistry, Sohag University, 82524 Sohag, Egypt

## Abstract

In the title mol­ecule, C_14_H_15_NO_3_, the ring system is essentially planar, with an r.m.s. deviation of 0.003 Å. The atoms of the ethane-1,2-diol group were refined as disordered over two sets of sites in a ratio of 0.815 (3):0.185 (3). The mol­ecular conformation is stabilized in part by an intra­molecular N—H⋯O hydrogen bond, which forms an *S*(6) ring. In the crystal, mol­ecules are connected by N—H⋯O and O—H⋯O hydrogen bonds, forming a two-dimensional network parallel to (100). The network also features weak C—H⋯O hydrogen bonds. Weak C—H⋯π inter­actions also observed.

## Related literature

For pharmaceutical and industrial applications of azomethines, see: Prakash & Adhikari (2011 ▶). For the effect of hydro­philicity on drug properties, see: Lin & Lu (1997 ▶). For standard bond lengths, see: Allen *et al.* (1987 ▶). For hydrogen-bond motifs, see: Bernstein *et al.* (1995 ▶).
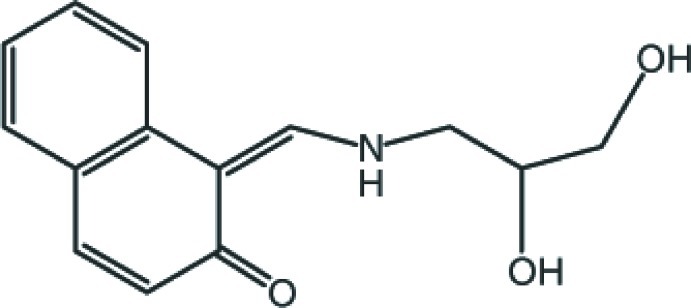


## Experimental

### 

#### Crystal data

C_14_H_15_NO_3_
*M* *_r_* = 245.27
Monoclinic, 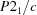

*a* = 23.452 (16) Å
*b* = 5.809 (4) Å
*c* = 8.739 (6) Å
β = 96.445 (7)°
*V* = 1183.0 (14) Å^3^
*Z* = 4
Mo *K*α radiation
μ = 0.10 mm^−1^
*T* = 100 K
0.27 × 0.14 × 0.01 mm

#### Data collection

Rigaku AFC12 (Right) diffractometer
Absorption correction: multi-scan (*CrystalClear-SM Expert*; Rigaku, 2012 ▶) *T*_min_ = 0.974, *T*_max_ = 0.999
8146 measured reflections
2650 independent reflections
2438 reflections with *I* > 2σ(*I*)
*R*_int_ = 0.025

#### Refinement

*R*[*F* ^2^ > 2σ(*F* ^2^)] = 0.058
*wR*(*F* ^2^) = 0.156
*S* = 1.11
2650 reflections
184 parameters
81 restraints
H-atom parameters constrained
Δρ_max_ = 0.67 e Å^−3^
Δρ_min_ = −0.26 e Å^−3^

### 

Data collection: *CrystalClear-SM Expert* (Rigaku, 2012 ▶); cell refinement: *CrystalClear-SM Expert*; data reduction: *CrystalClear-SM Expert*; program(s) used to solve structure: *SHELXS97* (Sheldrick, 2008 ▶); program(s) used to refine structure: *SHELXL97* (Sheldrick, 2008 ▶); molecular graphics: *PLATON* (Spek, 2009 ▶); software used to prepare material for publication: *WinGX* (Farrugia, 2012 ▶) and *PLATON*.

## Supplementary Material

Crystal structure: contains datablock(s) global, I. DOI: 10.1107/S1600536812051070/lh5572sup1.cif

Structure factors: contains datablock(s) I. DOI: 10.1107/S1600536812051070/lh5572Isup2.hkl

Supplementary material file. DOI: 10.1107/S1600536812051070/lh5572Isup3.cml

Additional supplementary materials:  crystallographic information; 3D view; checkCIF report

## Figures and Tables

**Table 1:** Hydrogen-bond geometry (Å, °) *Cg*1 and *Cg*2 are the centroids of the C1–C5/C10 and C5–C10 rings, respectively.

*D*—H⋯*A*	*D*—H	H⋯*A*	*D*⋯*A*	*D*—H⋯*A*
N1—H1⋯O1	0.88	1.87	2.560 (3)	135
N1—H1⋯O3*A* ^i^	0.88	2.56	3.166 (3)	127
O2*A*—H2*A*⋯O1^ii^	0.84	1.83	2.663 (3)	175
O3*A*—H3*A*⋯O2*A* ^iii^	0.84	1.91	2.744 (3)	169
C12—H12*B*⋯O1^iv^	0.99	2.60	3.174 (3)	117
C4—H4⋯*Cg*2^v^	0.95	2.79	3.491 (3)	132
C9—H9⋯*Cg*1^iv^	0.95	2.77	3.510 (3)	135

Symmetry codes: (i) 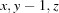; (ii) 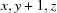; (iii) 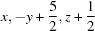; (iv) 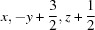; (v) 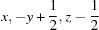.

**Fig. 1.**
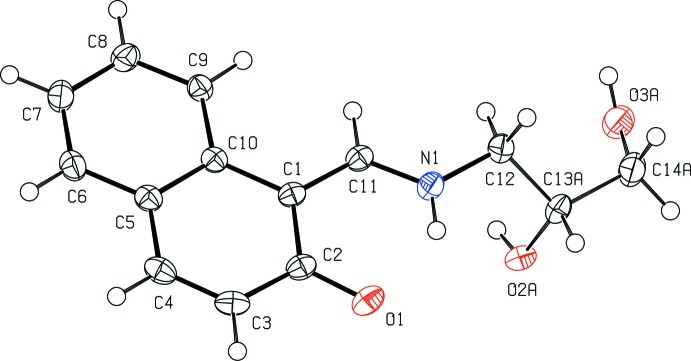
The molecular structure of the title compound with ellipsoids drawn at the 50% probability level. Only the major components of disorder are shown.

**Fig. 2.**
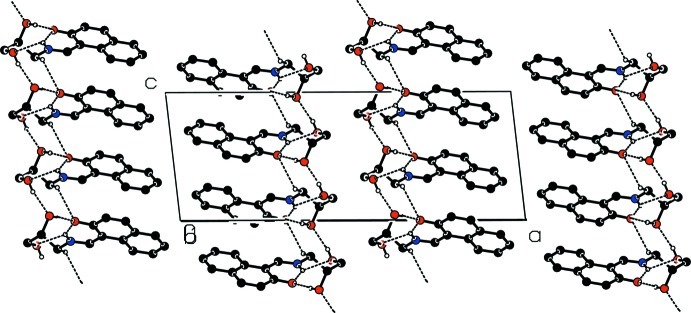
Crystal packing of (I) viewed along the b axis. Only the major component of disorder is shown. The hydrogen atoms not involved in the hydrogen bonds have been omitted for clarity.

## supplementary crystallographic information

### Comment

Azomethine compounds, which were named as Schiff's bases in 1864 are extensively incoporated in many pharmaceutical and food industry applications (Prakash & Adhikari, 2011). Elimination of excess drugs from the bloodstream or body is an essential process to protect against potential toxicity. In most cases the more hydrophilic drugs/pharmacophores are the more they are readily excreted by the kidneys in urine (Lin & Lu, 1997). The existance of conjugated double bonds and more hydroxylic groups in bioactive molecules increases not only their hydrophilicity but also the rate of their membrane absorption. Based on such facts we herein report the crystal structure of a potential bioactive hydrophilic azomethine derivative.

The molecluar structure of the title compound (I) is shown in Fig. 1. The naphthalene ring system (C1—C10) is essentially planar with an r.m.s. deviation of 0.003Å. The bond lengths (Allen *et al.*, 1987) and angles are within normal ranges. In the crystal, molecules are connected by N—H···O and O—H···O hydrogen bonds to form a two-dimensional network parallel to (100). The network is further stabilized by weak C—H···O hydrogen bonds. Weak C—H···π interactions also observed. The O—H groups of the minor component of disorder are not considered in the description of the hydrogen bonding.

### Experimental

A mixture of 1 mmol (172 mg) 2-hydroxynaphthalene-1-carbaldehyde and 1 mmol (91 mg) 3-aminopropane-1,2-diol in 40 ml ethanol was refluxed and monitored by TLC till completion after 12 h. On cooling of the reaction mixture at room temperature a quantitaive solid product was deposited, filtered and washed with cold ethanol. The crude product was crystallized from ethanol to afford x-ray quality yellow plates (m.p 505 K) in an excellent yield (90.6%) on a slow evaporation at room temperature for 24 h.

### Refinement

All H-atoms were placed in calculated positions and refined by using a riding model with O—H = 0.84 Å, N—H = 0.88 Å, C—H = 0.95 Å (aromatic), 0.99 Å (methylene) and 1.00 Å (methine), and with *U*_iso_(H) = 1.5*U*_eq_(O) for hydroxyl and *U*_iso_(H) = 1.2*U*_eq_(C) for the other atoms. The atoms of the ethane-1,2-diol group are disordered over two sets of sites with occupancies 0.815 (3) and 0.185 (3).

### Crystal data

**Table:**

C_14_H_15_NO_3_	*F*(000) = 520
*M**_r_* = 245.27	*D*_x_ = 1.377 Mg m^−^^3^
Monoclinic, *P*2_1_/*c*	Mo *K*α radiation, λ = 0.71075 Å
Hall symbol: -P 2ybc	Cell parameters from 2698 reflections
*a* = 23.452 (16) Å	θ = 2.4–27.6°
*b* = 5.809 (4) Å	µ = 0.10 mm^−^^1^
*c* = 8.739 (6) Å	*T* = 100 K
β = 96.445 (7)°	Sheet, yellow
*V* = 1183.0 (14) Å^3^	0.27 × 0.14 × 0.01 mm
*Z* = 4	

### Data collection

**Table:**

Rigaku AFC12 (Right) diffractometer	2650 independent reflections
Radiation source: Rotating Anode	2438 reflections with *I* > 2σ(*I*)
Detector resolution: 28.5714 pixels mm^-1^	*R*_int_ = 0.025
profile data from ω–scans	θ_max_ = 27.5°, θ_min_ = 3.5°
Absorption correction: multi-scan (*CrystalClear-SM Expert*; Rigaku, 2012)	*h* = −30→30
*T*_min_ = 0.974, *T*_max_ = 0.999	*k* = −7→7
8146 measured reflections	*l* = −11→9

### Refinement

**Table:**

Refinement on *F*^2^	Primary atom site location: structure-invariant direct methods
Least-squares matrix: full	Secondary atom site location: difference Fourier map
*R*[*F*^2^ > 2σ(*F*^2^)] = 0.058	Hydrogen site location: inferred from neighbouring sites
*wR*(*F*^2^) = 0.156	H-atom parameters constrained
*S* = 1.11	*w* = 1/[σ^2^(*F*_o_^2^) + (0.0733*P*)^2^ + 0.7284*P*] where *P* = (*F*_o_^2^ + 2*F*_c_^2^)/3
2650 reflections	(Δ/σ)_max_ < 0.001
184 parameters	Δρ_max_ = 0.67 e Å^−^^3^
81 restraints	Δρ_min_ = −0.26 e Å^−^^3^

### Special details

**Table:**

Geometry. Bond distances, angles *etc*. have been calculated using the rounded fractional coordinates. All su's are estimated from the variances of the (full) variance-covariance matrix. The cell e.s.d.'s are taken into account in the estimation of distances, angles and torsion angles
Refinement. Refinement on *F*^2^ for ALL reflections except those flagged by the user for potential systematic errors. Weighted *R*-factors *wR* and all goodnesses of fit *S* are based on *F*^2^, conventional *R*-factors *R* are based on *F*, with *F* set to zero for negative *F*^2^. The observed criterion of *F*^2^ > σ(*F*^2^) is used only for calculating -*R*-factor-obs *etc*. and is not relevant to the choice of reflections for refinement. *R*-factors based on *F*^2^ are statistically about twice as large as those based on *F*, and *R*-factors based on ALL data will be even larger.

### Fractional atomic coordinates and isotropic or equivalent isotropic displacement parameters (Å^2^)

**Table:**

	*x*	*y*	*z*	*U*_iso_*/*U*_eq_	Occ. (<1)
O1	0.30508 (5)	0.3921 (2)	0.50408 (16)	0.0291 (4)	
O2A	0.37874 (6)	1.0576 (3)	0.45655 (17)	0.0239 (5)	0.815 (3)
O3A	0.42610 (7)	1.3999 (3)	0.6847 (2)	0.0333 (5)	0.815 (3)
N1	0.32519 (6)	0.7647 (3)	0.65713 (18)	0.0236 (4)	
C1	0.23196 (7)	0.5877 (3)	0.62006 (19)	0.0194 (5)	
C2	0.25288 (7)	0.4010 (3)	0.5343 (2)	0.0222 (5)	
C3	0.21329 (8)	0.2228 (3)	0.4789 (2)	0.0252 (5)	
C4	0.15774 (8)	0.2281 (3)	0.5059 (2)	0.0249 (5)	
C5	0.13503 (7)	0.4101 (3)	0.59118 (19)	0.0211 (5)	
C6	0.07683 (7)	0.4098 (3)	0.6178 (2)	0.0255 (5)	
C7	0.05463 (7)	0.5851 (3)	0.6992 (2)	0.0277 (5)	
C8	0.09095 (7)	0.7645 (3)	0.7572 (2)	0.0257 (5)	
C9	0.14798 (7)	0.7680 (3)	0.7335 (2)	0.0211 (5)	
C10	0.17201 (7)	0.5925 (3)	0.64858 (19)	0.0191 (5)	
C11	0.27077 (7)	0.7636 (3)	0.67604 (19)	0.0205 (5)	
C12	0.36595 (7)	0.9416 (3)	0.7169 (2)	0.0252 (5)	
C13A	0.40851 (18)	0.9966 (7)	0.6026 (5)	0.0238 (8)	0.815 (3)
C14A	0.4511 (3)	1.1822 (11)	0.6651 (7)	0.0321 (10)	0.815 (3)
O2B	0.4314 (4)	0.8181 (15)	0.5357 (10)	0.043 (2)*	0.185 (3)
O3B	0.4728 (6)	1.153 (3)	0.8021 (16)	0.082 (4)*	0.185 (3)
C13B	0.4028 (10)	1.008 (3)	0.591 (2)	0.028 (3)*	0.185 (3)
C14B	0.4461 (17)	1.192 (5)	0.653 (3)	0.032 (3)*	0.185 (3)
H4	0.13300	0.10710	0.46700	0.0300*	
H6	0.05260	0.28710	0.57900	0.0310*	
H7	0.01530	0.58460	0.71590	0.0330*	
H8	0.07590	0.88560	0.81380	0.0310*	
H9	0.17170	0.89070	0.77500	0.0250*	
H11	0.25630	0.88810	0.73060	0.0250*	
H12A	0.38710	0.88780	0.81480	0.0300*	0.815 (3)
H12B	0.34480	1.08320	0.73870	0.0300*	0.815 (3)
H13A	0.43090	0.85330	0.58790	0.0290*	0.815 (3)
H14A	0.47050	1.13040	0.76570	0.0380*	0.815 (3)
H14B	0.48070	1.19870	0.59380	0.0380*	0.815 (3)
H1	0.33820	0.65070	0.60460	0.0280*	
H2A	0.35380	1.15760	0.46900	0.0360*	0.815 (3)
H3	0.22650	0.09830	0.42190	0.0300*	
H3A	0.41610	1.40930	0.77390	0.0500*	0.815 (3)
H2B	0.45860	0.77900	0.60140	0.0640*	0.185 (3)
H3B	0.50210	1.07090	0.79770	0.1230*	0.185 (3)
H12C	0.39200	0.87960	0.80430	0.0300*	0.185 (3)
H12D	0.34520	1.07520	0.75410	0.0300*	0.185 (3)
H13B	0.37720	1.07550	0.50300	0.0330*	0.185 (3)
H14C	0.47610	1.20420	0.58210	0.0390*	0.185 (3)
H14D	0.42600	1.34240	0.65150	0.0390*	0.185 (3)

### Atomic displacement parameters (Å^2^)

**Table:**

	*U*^11^	*U*^22^	*U*^33^	*U*^12^	*U*^13^	*U*^23^
O1	0.0279 (7)	0.0284 (7)	0.0328 (8)	0.0057 (5)	0.0117 (5)	−0.0005 (5)
O2A	0.0279 (8)	0.0233 (8)	0.0218 (8)	0.0035 (6)	0.0084 (6)	−0.0008 (6)
O3A	0.0305 (9)	0.0319 (9)	0.0388 (10)	−0.0010 (7)	0.0096 (7)	−0.0029 (7)
N1	0.0210 (7)	0.0253 (7)	0.0253 (8)	−0.0004 (6)	0.0056 (6)	0.0002 (6)
C1	0.0219 (8)	0.0192 (8)	0.0174 (8)	0.0022 (6)	0.0038 (6)	0.0024 (6)
C2	0.0258 (8)	0.0234 (8)	0.0182 (8)	0.0032 (6)	0.0054 (6)	0.0038 (6)
C3	0.0363 (10)	0.0202 (8)	0.0193 (9)	0.0047 (7)	0.0044 (7)	−0.0015 (6)
C4	0.0339 (9)	0.0191 (8)	0.0209 (9)	−0.0028 (7)	−0.0003 (7)	−0.0004 (6)
C5	0.0246 (8)	0.0212 (8)	0.0172 (8)	−0.0013 (6)	0.0008 (6)	0.0025 (6)
C6	0.0240 (8)	0.0267 (9)	0.0252 (9)	−0.0056 (7)	−0.0002 (7)	0.0005 (7)
C7	0.0208 (8)	0.0346 (10)	0.0274 (10)	−0.0030 (7)	0.0021 (6)	0.0023 (8)
C8	0.0233 (8)	0.0271 (9)	0.0272 (9)	0.0042 (7)	0.0046 (7)	−0.0018 (7)
C9	0.0206 (8)	0.0201 (8)	0.0222 (8)	−0.0007 (6)	0.0009 (6)	−0.0013 (6)
C10	0.0212 (8)	0.0194 (8)	0.0163 (8)	0.0006 (6)	0.0006 (6)	0.0029 (6)
C11	0.0219 (8)	0.0215 (8)	0.0186 (8)	0.0022 (6)	0.0040 (6)	0.0033 (6)
C12	0.0212 (8)	0.0295 (9)	0.0251 (9)	−0.0027 (7)	0.0032 (6)	−0.0002 (7)
C13A	0.0181 (13)	0.0256 (12)	0.0287 (14)	0.0030 (9)	0.0065 (12)	0.0002 (9)
C14A	0.0221 (19)	0.0333 (15)	0.0417 (19)	−0.0031 (13)	0.0076 (12)	−0.0039 (13)

### Geometric parameters (Å, º)

**Table:**

O1—C2	1.282 (2)	C9—C10	1.415 (3)
O2A—C13A	1.429 (5)	C12—C13B	1.52 (2)
O2B—C13B	1.41 (2)	C12—C13A	1.523 (5)
O3A—C14A	1.412 (7)	C13A—C14A	1.528 (8)
O3B—C14B	1.40 (3)	C13B—C14B	1.53 (4)
O2A—H2A	0.8400	C3—H3	0.9500
O2B—H2B	0.8400	C4—H4	0.9500
O3A—H3A	0.8400	C6—H6	0.9500
O3B—H3B	0.8400	C7—H7	0.9500
N1—C12	1.460 (3)	C8—H8	0.9500
N1—C11	1.305 (2)	C9—H9	0.9500
N1—H1	0.8800	C11—H11	0.9500
C1—C11	1.418 (3)	C12—H12C	0.9900
C1—C2	1.436 (3)	C12—H12D	0.9900
C1—C10	1.455 (3)	C12—H12A	0.9900
C2—C3	1.438 (3)	C12—H12B	0.9900
C3—C4	1.350 (3)	C13A—H13A	1.0000
C4—C5	1.430 (3)	C13B—H13B	1.0000
C5—C10	1.424 (3)	C14A—H14A	0.9900
C5—C6	1.410 (3)	C14A—H14B	0.9900
C6—C7	1.377 (3)	C14B—H14C	0.9900
C7—C8	1.404 (3)	C14B—H14D	0.9900
C8—C9	1.376 (3)		
			
C13A—O2A—H2A	109.00	C3—C4—H4	119.00
C13B—O2B—H2B	109.00	C5—C4—H4	119.00
C14A—O3A—H3A	109.00	C7—C6—H6	120.00
C14B—O3B—H3B	109.00	C5—C6—H6	120.00
C11—N1—C12	124.65 (16)	C6—C7—H7	120.00
C12—N1—H1	118.00	C8—C7—H7	120.00
C11—N1—H1	118.00	C9—C8—H8	119.00
C2—C1—C10	119.81 (15)	C7—C8—H8	119.00
C10—C1—C11	121.46 (15)	C10—C9—H9	119.00
C2—C1—C11	118.72 (15)	C8—C9—H9	119.00
C1—C2—C3	118.30 (15)	C1—C11—H11	118.00
O1—C2—C1	121.88 (15)	N1—C11—H11	118.00
O1—C2—C3	119.81 (16)	N1—C12—H12C	110.00
C2—C3—C4	121.61 (16)	N1—C12—H12A	109.00
C3—C4—C5	122.07 (16)	N1—C12—H12B	109.00
C6—C5—C10	120.34 (15)	C13A—C12—H12B	109.00
C4—C5—C6	120.60 (16)	H12A—C12—H12B	108.00
C4—C5—C10	119.06 (15)	C13B—C12—H12C	107.00
C5—C6—C7	120.88 (16)	C13B—C12—H12D	112.00
C6—C7—C8	119.08 (15)	H12C—C12—H12D	108.00
C7—C8—C9	121.13 (16)	N1—C12—H12D	110.00
C8—C9—C10	121.29 (16)	C13A—C12—H12A	109.00
C5—C10—C9	117.27 (15)	O2A—C13A—H13A	108.00
C1—C10—C9	123.57 (15)	C14A—C13A—H13A	108.00
C1—C10—C5	119.15 (15)	C12—C13A—H13A	108.00
N1—C11—C1	123.96 (16)	O2B—C13B—H13B	108.00
N1—C12—C13B	108.8 (7)	C12—C13B—H13B	108.00
N1—C12—C13A	111.4 (2)	C14B—C13B—H13B	108.00
O2A—C13A—C14A	112.2 (4)	H14A—C14A—H14B	108.00
O2A—C13A—C12	110.3 (3)	C13A—C14A—H14B	109.00
C12—C13A—C14A	111.3 (4)	O3A—C14A—H14A	109.00
C12—C13B—C14B	109.2 (15)	O3A—C14A—H14B	109.00
O2B—C13B—C14B	110 (2)	C13A—C14A—H14A	109.00
O2B—C13B—C12	112.4 (12)	O3B—C14B—H14C	109.00
O3A—C14A—C13A	114.3 (5)	O3B—C14B—H14D	108.00
O3B—C14B—C13B	115 (2)	C13B—C14B—H14C	109.00
C2—C3—H3	119.00	C13B—C14B—H14D	108.00
C4—C3—H3	119.00	H14C—C14B—H14D	107.00
			
C12—N1—C11—C1	−178.57 (16)	C3—C4—C5—C6	−179.74 (17)
C11—N1—C12—C13A	−141.4 (2)	C4—C5—C6—C7	−179.69 (16)
C10—C1—C2—O1	178.69 (16)	C4—C5—C10—C9	−179.45 (16)
C11—C1—C2—C3	179.61 (16)	C10—C5—C6—C7	0.1 (3)
C10—C1—C2—C3	0.0 (2)	C4—C5—C10—C1	−0.5 (2)
C11—C1—C2—O1	−1.7 (3)	C6—C5—C10—C9	0.8 (2)
C11—C1—C10—C5	−179.31 (16)	C6—C5—C10—C1	179.68 (16)
C11—C1—C10—C9	−0.5 (3)	C5—C6—C7—C8	−0.6 (3)
C2—C1—C11—N1	−1.4 (3)	C6—C7—C8—C9	0.2 (3)
C10—C1—C11—N1	178.20 (16)	C7—C8—C9—C10	0.7 (3)
C2—C1—C10—C5	0.3 (2)	C8—C9—C10—C5	−1.1 (3)
C2—C1—C10—C9	179.17 (16)	C8—C9—C10—C1	180.00 (16)
O1—C2—C3—C4	−178.79 (17)	N1—C12—C13A—O2A	54.6 (3)
C1—C2—C3—C4	−0.1 (3)	N1—C12—C13A—C14A	179.9 (3)
C2—C3—C4—C5	−0.2 (3)	O2A—C13A—C14A—O3A	58.8 (5)
C3—C4—C5—C10	0.5 (3)	C12—C13A—C14A—O3A	−65.4 (5)

### Hydrogen-bond geometry (Å, º)

Cg1 and Cg2 are the centroids of the C1–C5/C10 and C5–C10 rings, respectively.

**Table:**

*D*—H···*A*	*D*—H	H···*A*	*D*···*A*	*D*—H···*A*
N1—H1···O1	0.88	1.87	2.560 (3)	135
N1—H1···O3*A*^i^	0.88	2.56	3.166 (3)	127
O2*A*—H2*A*···O1^ii^	0.84	1.83	2.663 (3)	175
O3*A*—H3*A*···O2*A*^iii^	0.84	1.91	2.744 (3)	169
C12—H12*B*···O1^iv^	0.99	2.60	3.174 (3)	117
C4—H4···*Cg*2^v^	0.95	2.79	3.491 (3)	132
C9—H9···*Cg*1^iv^	0.95	2.77	3.510 (3)	135

Symmetry codes: (i) *x*, *y*−1, *z*; (ii) *x*, *y*+1, *z*; (iii) *x*, −*y*+5/2, *z*+1/2; (iv) *x*, −*y*+3/2, *z*+1/2; (v) *x*, −*y*+1/2, *z*−1/2.
